# Quantitative Evaluation of the Eco-Environment in a Coalfield Based on Multi-Temporal Remote Sensing Imagery: A Case Study of Yuxian, China

**DOI:** 10.3390/ijerph16030511

**Published:** 2019-02-12

**Authors:** Xue Wang, Kun Tan, Kailei Xu, Yu Chen, Jianwei Ding

**Affiliations:** 1 Key Laboratory for Land Environment and Disaster Monitoring of NASG, China University of Mining and Technology, Xuzhou 221116, China; wx_cumt@yeah.net (X.W.); XKL_1992@126.com (K.X.); 2 Key Laboratory of Geographic Information Science (Ministry of Education), East China Normal University, Shanghai 200241, China; 3 MEIHANG Remote Sensing Information Co., Ltd, Xi’an 710199, China; 4 The Second Surveying and Mapping Institute of Hebei, Shijiazhuang 050037, China; djw19721122@163.com

**Keywords:** multi-temporal remote sensing imagery, land cover, analytic hierarchy process, ecological footprint, coalfield

## Abstract

With the exploitation of coalfields, the eco-environment around the coalfields can become badly damaged. To address this issue, “mine greening” has been proposed by the Ministry of Land and Resources of China. The sustainable development of mine environments has now become one of the most prominent issues in China. In this study, we aimed to make use of Landsat 7 ETM+ and Landsat 8 OLI images obtained between 2005 and 2016 to analyze the eco-environment in a coalfield. Land cover was implemented as the basic evaluation factor to establish the evaluation model for the eco-environment. Analysis and investigation of the eco-environment in the Yuxian coalfield was conducted using a novel evaluation model, based on the biological abundance index, vegetation coverage index, water density index, and natural geographical factors. The weight of each indicator was determined by an analytic hierarchy process. Meanwhile, we also used the classic ecological footprint to calculate the ecological carrying capacity in order to verify the effectiveness of the evaluation model. Results showed that the eco-environment index illustrated a slowly increasing tendency over the study period, and the ecological quality could be considered as “good”. The results of the evaluation model showed a strong correlation with the ecological carrying capacity with a correlation coefficient of 0.9734. In conclusion, the evaluation method is a supplement to the time-series quantitative evaluation of the eco-environment, and also helps us to explore the eco-environment in the mining area.

## 1. Introduction

China has been experiencing rapid urban development with the economic reform taking place over the past 20 years. Coal has become one of the most important power sources and accounts for about 70% of China’s primary energy consumption [1]. The original balance of the rock stratum is inevitably changed after the exploitation of coal resources. The collapse of the Earth’s surface caused by mining activities can trigger a series of environmental disasters [2]. To address this issue, “mine greening” has been proposed by the Ministry of Land and Resources of China [3], which is a new idea for mineral resources management. Huang [4] constructed the evaluation index system for “mine greening” based on legitimate mining, efficient utilization, cleaner production and standardized management. Compared with traditional field measurements, remote sensing technology has many superiorities, including the large observation range, land cover mapping on fine-scale, the regular revisit period, and a reduced requirement for manual labor, which makes it capable for the environment monitoring of mining areas [5].

Remote sensing technology has been used in the fields of environmental monitoring [6,7,8], land use and land cover (LUCC) change [9,10,11], agricultural applications [12,13,14], military applications [15], and disaster control [16]. As for the research on environment monitoring of mining areas, Singh et al. [17] monitored the impact of coal mining and thermal power industry on land use pattern by remote sensing data. Townsend et al. [18] utilized a Landsat time series from 1976 to 2006 to explore the dominate driver of LUCC change in the Eastern U.S. Karan et al. [19] verified the effectiveness of the vegetation factors to indicate the reclamation successful of coal mine degraded lands based on the remote sensing imagery. Chen et al. [5] checked almost all the available literature about LUCC classification in open-pit mine areas (LCCMA) based on ISI and Google scholar and summarized fine-scale LCCMA with the accuracy limited issues, the feature extraction methods and the developing directions.

Many studies have shown that information extracted from remote sensing images can be applied to evaluate the condition of the environment. In previous researches, remote sensing images have been applied to environmental monitoring in the aspects of ecological vulnerability [20,21], ecological sensitivity [21,22], net primary productivity (NPP) [23,24], ecological footprint [25,26], and ecological carrying capacity. However, there are no unified standards for the selection of evaluation indicators. Most of previous evaluation studies of eco-environment were emphasized on some particular research fields such as wastewater and petroleum-contaminated sites [27,28]. The multi synthesis analysis and unitary assessment were rarely found on mining area. The eco-environmental quality index [29] has become an important measure for judging environmental indicators. Meanwhile, the combination of strategic analysis and remote sensing has become the main approach in the field of environmental evaluation. The analytic hierarchy process (AHP), which was proposed by Saaty in 1980, can be used to determine the weights of the indicators, and features a hierarchical decision model and simple operation [30,31]. By combining AHP and remote sensing technology, we aimed at constructing the integrated evaluation systems from the aspect of mining area. The vegetation coverage, water environment, biological abundance and natural geography are considered by taking into account both HJ/T192-2006 and prophase field investigation.

In this study, a novel coalfield evaluation system is presented, which incorporates the biological abundance index (BAI), the vegetation coverage index (VCI), the water density index (WDI), and the natural geographical factors (NGF). According to the characteristics of coalfields, supervised classification based on maximum likelihood algorithm with Landsat images of the Yuxian coalfield is used for extraction of the land cover data and the regions of interest (ROI) are chosen based on field survey data and visual interpretation for supervising the training. The integration of information extracted from the multi-temporal Landsat images and other auxiliary data achieved time-series environmental quality evaluation through the AHP method. In order to verify the effectiveness of the evaluation model, the ecological carrying capacity of the ecological footprint (as proposed by Rees et al. [32]) was applied. The environmental quality evaluation of coalfields is of significance to the natural environment and sustainable socio-economic development.

## 2. Study Area

**Figure 1:**
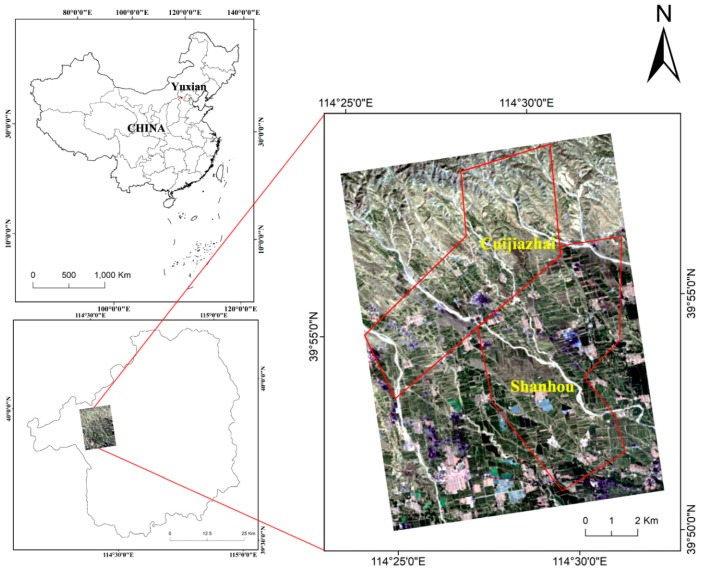
Geographical location of the coalfield and study area. (The remote sensing imagery resource is OLI and the band combination is 4(R) 3(G) 2(B)).

**Figure 2:**
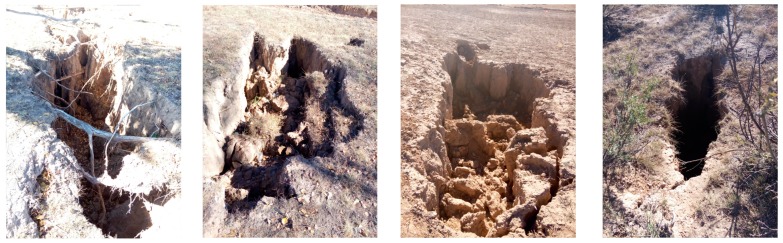
Ground fissure and collapse in the study area.

The Shanhou and Cuijiazhai coalfield is located between 114°24′40″E–114°32′30″E and 39°50′54″N–39°58′25″N in Yuxian County, Zhangjiakou City, Hebei Province, China, shown in Figure 1. This area belongs to the warm temperate continental monsoon climate zone, and the annual precipitation is between 380 and 683 mm. The monsoon climate generates four distinct seasons in Yuxian County. A cool summer, changeable autumn, and lower temperature in winter are the characteristics of the climate in Yuxian County. The temperature in winter can occasionally reach −20 °C. Coal industry is the most important industry in Yuxian County, resulted from many superiorities of this coalfield, such as the shallow burial depth, low slope, and low water inflow in the interior of the coalfield. However, some problems have arisen with the exploitation of the coalfield, including ground fissures and collapse (see Figure 2). The mining exploitation has also increased the likelihood of geological disasters. Moreover, sustained precipitation may further aggravate the geological disasters and cause casualties or property loss.

## 3. Materials and Methods

### 3.1. Data Sources and Processing

A complementary set of land-cover observations were provided by the Operational Land Imager (OLI) and Enhanced Thematic Mapper Plus (ETM+) sensors. The Landsat Earth resources satellite system was the first system designed to provide near global coverage of the Earth’s surface on a regular and predictable basis [33]. The 30-m spatial resolution of these sensors makes it possible to extract surface information such as land cover, vegetation distribution, and urban impervious surfaces. With the data continuity of Landsat over the past decades, Landsat data based time-series analysis allows for an effective characterization of the temporal and spatial variation of the eco-environment [34,35,36]. The images used in this study were downloaded from the U. S. Geological Survey (USGS) (http://earthexplorer.usgs.gov/) and the Geospatial Data Cloud (http://www.gscloud.cn/). The acquisition period of these data was from 2005 to 2016. Multi-temporal analysis of Landsat imagery presents challenges in terms of data availability. The L1TP products were utilized in our work and the atmospheric correction was done by FLAASH model which has been demonstrated to be sufficiently accurate for the image classification [37,38,39,40].

The images in 2010 were deficient because of the heavy cloud cover, so we skipped this year. Finally, we collected 44 scenes, covering the March, July, September, and December of each year. Given the data availability, we replaced the data which were not available with data from adjacent months. The data list is listed in Table 1.

Because the original remote sensing data had only been processed with rough radiometric correction and geometric correction, it was necessary to preprocess the data, including clipping, re-sampling, and projection conversion. The Landsat calibration module and the Fast Line-of sight Atmosphere Analysis of Spectral Hypercube (FLAASH) algorithm provided in ENVI5.1 software (Harris Geospatial Solutions, Broomfield, CO, USA) was used to achieve atmospheric correction. The geometric registration root-mean-square (RMS) deviation was found to be less than 0.5 pixels, when compared with field investigation. Finally, the images were converted to the World Geodetic System-1984 (WGS-84) coordinate system and Universal Transverse Mercator (UTM) projection. The ETM+ sensor Scan Line Corrector (SLC) malfunctioned in May 2003, so the captured images contained missing data strips after 2003. We therefore applied the integrated algorithm proposed by Zeng et al. [41] to repair the missing data strips.

In addition, auxiliary data were also integrated into the environmental quality evaluation. Meteorological data were provided by the China Meteorological Data Service Center of the China Meteorological Administration (http://data.cma.cn/). Statistical data were acquired from Statistical Yearbooks of China’s counties and cities, which were downloaded from the National Bureau of Statistics of China (http://www.stats.gov.cn/tjsj/).

### 3.2. Research Methods

#### 3.2.1. Construction of the Environmental Quality Evaluation Index System

The evaluation of environmental quality is a multi-disciplinary and multi-technology task, which combines ecology, statistics, earth observation, etc. After comprehensive consideration of the data availability and the natural conditions of the Yuxian County coalfield, the index system was established based on the “Technical specifications of eco-environment evaluation (for trial implementation)” (HJ/T192-2006). Specifically, the individual indices were made up of the BAI, VCI, WDI, and NGF [42,43]. The main influencing factors of these four major aspects were cultivated land, water body, grassland, building land, bare land, digital elevation model (DEM), precipitation, vegetation condition, and surface runoff area, the first five of which were obtained by classification of Landsat imagery.

The impact of mining activities on the surrounding environment is mainly manifested in the change of land cover. On the other hand, land cover is an important environmental variable and a key input parameter for ecological models [34]. The ROI is divided into training set and test set when the supervised classification is performed. The overall accuracy of the classification in this study was over 85% verified by the test set, which can satisfy the demand of eco-environment evaluation. The accuracy assessment on 2016 as the example is listed in Table 2. In addition, vegetation coverage is also a key parameter for environmental evaluation, as vegetation plays an important role in soil and water conservation, environmental purification, and oxygen exchange. Due to the fact that abnormal precipitation and the topographic gradient are both significant factors which can cause geological disasters in mining areas, both of these factors were selected as representative indices.

#### 3.2.2. Weight Determination Based on a Multi-Criteria Decision-Making Technique

The weight is a quantitative coefficient of the importance distribution of the impact factor of the research object [44]. AHP is a multi-objective decision analysis method which combines quantitative analysis with qualitative analysis [31]. It can be combined with fuzzy mathematics to establish a membership function for a correlation analysis. When the indicators are few in number and a high evaluation accuracy is required, AHP can be regarded as a good choice. The assigned weight is normalized on the basis of Saaty’s scale, considering two themes and classes at a time, on the basis of their relative importance, to determine the eco-environmental index (EI). Thereafter, pair-wise comparison matrices of the weights assigned to the different thematic layers and their individual classes are constructed using Saaty’s AHP, and the weights are normalized by an eigenvector approach. The consistency ratio (CR) is then calculated to examine the normalized weights of the various thematic layers and their individual classes, as per the recommendation of Saaty [28]. The following steps are carried out to compute the CR of the various thematic layers and their individual classes.

(1) Hierarchy Model Construction

**Figure 3:**
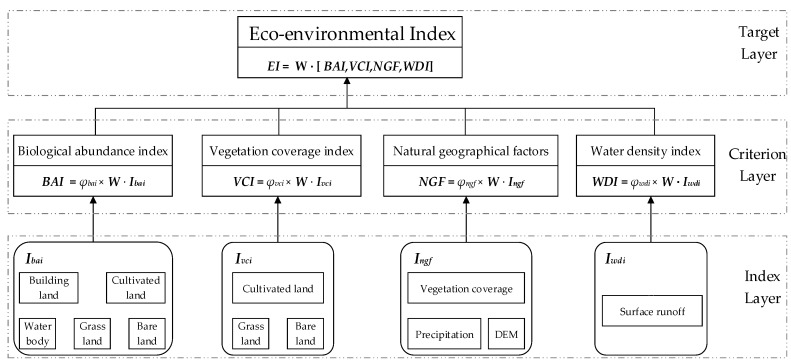
The Hierarchy model construction.

The HJ/T192-2006 was promulgated by the national environment protection bureau of China which specified the eco-environmental assessment indexes and the corresponding computing methods. In HJ/T192-2006, EI is utilized to evaluate the regional eco-environment quality with a range of 0 to 100 comprehensively. Combining the indexes given by HJ/T192-2006 and the field investigation, we used the BAI, VCI, WDI and NGF as the indicators of criterion layer to established the hierarchy model. This lays an important foundation for the environmental quality evaluation in the coalfield. The hierarchy model is shown in Figure 3.

The computing methods of all indicators in the index layer are shown in Table 3, the area of different land use is obtained from the post-classification statistics. The weight W and the normalization coefficient of each indicator φ are introduced in next section.

(2) Construction of Pair-Wise Comparison Matrices

Pair-wise comparison matrices are used to compare the importance of two indices. The importance of the pair-wise comparison judgment is shown in Table 4. The pair-wise comparison matrix A is built as follows:

(1) $$A=\left[\begin{array}{cccc} a_{11}^{\prime } & a_{12}^{\prime } & \cdots & a_{1n}^{\prime }\\ a_{21}^{\prime } & a_{22}^{\prime } & \cdots & a_{2n}^{\prime }\\ \vdots & \vdots & \ddots & \vdots \\ a_{n1}^{\prime } & a_{n2}^{\prime } & \cdots & a_{nn}^{\prime } \end{array}\right],\;a_{ij}^{\prime }=\frac{a_{ij}}{\sum_{i=1}^{n}a_{ij}}\;\mathrm{for}\;i,j=1,2,\ldots ,n$$

where *n* represents the number of evaluation criteria considered, each $a_{ij}$ of the matrix A represents the importance of the ith criterion relative to the jth criterion. If A > 1, then the ithcriterion is more important than the jth criterion, while if $a_{ij}$ < 1, then the ith criterion is less important than the jth criterion. Moreover, the $a_{ij}$ and $a_{ji}$ satisfy the reciprocal constraint. $a_{ij}^{\prime }$ denotes the normalized result by column. The upper half and lower half of the matrix diagonal are reciprocal to each other, therefore matrix A is a reciprocal matrix.

(3) Calculation of the Weight Vector

The eigenvalue and the eigenvector are calculated as follows:

(2) $$W=\left[\begin{array}{c} w_{1}\\ w_{2}\\ \vdots \\ w_{n} \end{array}\right]\;\mathrm{and}\;w_{i}=\frac{\sum_{j=1}^{n}a_{ij}^{\prime }}{n}\;\mathrm{for}\;i,\;j=1,2,3,\ldots ,n$$

(3) $$W^{\prime }=AW=\left[\begin{array}{c} w_{1}^{\prime }\\ w_{2}^{\prime }\\ \vdots \\ w_{n}^{\prime } \end{array}\right]$$

(4) $$\lambda_{max}=\frac{1}{n}\left(\frac{w_{1}^{\prime }}{w_{1}}+\frac{w_{2}^{\prime }}{w_{2}}+\ldots \ldots +\frac{w_{n}^{\prime }}{w_{n}}\right)$$

where W is the criteria weight vector, $W^{\prime }$ is utilized to calculate the average eigenvalue of the pair-wise comparison matrix $\lambda_{max}$.

(4) Consistency Inspection of the Judgment Matrix

For the judgment matrix, a consistency method is applied to test the reliability. If the pair-wise comparison matrix deviates from the consistency, the reliability of the results will be lower. To judge the uncertainty, Saaty’s consistency index (CI) is used, which is calculated using Equation (5):

(5) $$\;CI=\;\frac{\;\lambda_{max}-n}{n-1}$$

where *n* is the number of indices.

CR is a measurement of consistency of the pair-wise comparison matrix, and is calculated using Equation (6):

(6) $$\;CR=\frac{CI}{RI}$$

where RI is the ratio index. The standard values of RI are shown in Table 5. The CR is acceptable if CR ≤ 0.1; otherwise, we re-evaluate the corresponding weights to avoid inconsistency. Practically, 0.00001 was utilized as substitute for zero in the calculation of CR.

#### 3.2.3. Ecological Carrying Capacity Calculation Based on the Ecological Footprint

The ecological footprint refers to the various types of resources meeting the daily consumption of a certain human population and the area of ecologically productive land necessary for assimilating various types of associated domestic waste [46,47]. The ecological carrying capacity is calculated from the perspective of land supply based on quantification of a group of indices regarding the area of ecologically productive land [48]. The computational equation for the ecological carrying capacity is as follows [49]:

(7) $$ECC=N\cdot ecc=N\cdot \left(1-12\%\right)\cdot \sum \gamma \cdot y\cdot a_{i}$$

where *ECC* represents the total ecological carrying capacity; *N* represents the total population of the research area; *ecc* is the per capita ecological carrying capacity; 12% represents the 12% land area deducted from the ecological supply for protection of the biological productivity; *γ* is the equivalence factor; *y* is the yield factor; *i* is the area of biologically productive land required; and *a_i_* is the per capita area of the productive land of *i*.

The equivalence factor and the yield factor have been given by WWF on a big-scale and are not suitable on the regional scale. We utilized the equivalence factor and the yield factor for Hebei province which are calculated based on NPP [50], because the provincial ecological footprint model based on NPP can reflect the actual land productivity in the region of a province or city. In this way, the ecological footprint can be of practical value in medium- and small-scale regions. The relevant parameters are shown in Table 6.

#### 3.2.4. Comprehensive Evaluation Model Construction

Considering the reality of a coalfield, we adopted the comprehensive indices approach to calculate the final score of environmental quality. This approach not only is based on a clear principle, but also emphasizes the integrality and objectivity of the evaluation. The “integral” refers that the hierarchical structure is integral, each layer has its own calculational criterion and between layers the connection weights are under control by CR. The “objective” refers that the indicators are objective [51]. Based on the four main indicators and the weight sorting supported by HJ/T192-2006, the evaluation model of environmental quality is as follows:

(8) $$EI=V_{BAI}*w_{cl1}+V_{VCI}*w_{cl2}+V_{NGF}*w_{cl3}+V_{WDI}*w_{cl4}$$

where EI is the eco-environmental index, $w_{cli}$ denotes the weight of the *i*th indicator in criterion layer. $V_{BAI}$, $V_{VCI}$, $V_{NGF}$ and $V_{WDI}$ denote the values of the indicators in criterion layer respectively. The average value of EI in the four quarters of each year is used as the final EI of the year to exclude the influence of contingency.

During the calculation of the $V_{BAI}$, $V_{VCI}$, $V_{NGF}$ and $V_{WDI}$, the normalization coefficient of each indicator φ is utilized to guarantee the score of each indicator is less than or equal to 100:

(9) $$V_{BAI}=\varphi_{BAI}\times \left(V_{Water\;body}*w_{il-bai1}+V_{Building\;land}*w_{il-bai2}+V_{Cultivated\;land}*w_{il-bai3}\;+V_{Grass\;land}*w_{il-bai4}+V_{Bare\;land}*w_{il-bai5}\right)$$

(10) $$V_{VCI}=\varphi_{VCI}\times \left(V_{Cultivated\;land}*w_{il-vci1}+V_{Grass\;land}*w_{il-vci2}+V_{Bare\;land}*w_{il-vci3}\right)$$

(11) $$V_{NGF}=\varphi_{NGF}\times (V_{Precipitation}*w_{il-ngf1}+V_{DEM}*w_{il-ngf2}+V_{Vegetation\;coverage}*w_{il-ngf3})$$

(12) $$V_{WDI}=\varphi_{WDI}\times \left(V_{Surface\;runoff}*w_{il-wdi}\right)$$

where $w_{il-bai}$, $w_{il-vci}$ $w_{il-ngf}$ and $w_{il-wdi}$ represent the weights of index layer for BAI, VCI, NGF and WDI respectively. Taking the maximum value among the middle values of each indicator, the normalization coefficient of each evaluation indicator is calculated. The score of each indicator is less than or equal to 100, and the final EI value is also between 0 and 100. The normalization coefficient $\varphi_{BAI}$, $\varphi_{VCI}$, $\varphi_{NGF}$ and $\varphi_{WDI}$ are calculated using Equation (13):

(13) $$\varphi_{i}=\;\frac{100}{I_{max}}$$

where *φ_i_* is the normalization coefficient of each indicator; and *I_max_* represents the maximum value among the middle values of each indicator. The *I_max_* of each indicator is as follows:

(14) $$I_{max-BAI}=\underset{time}{\max}{\left(V_{Water\;body}*w_{il-bai1}+V_{Building\;land}*w_{il-bai2}+V_{Cultivated\;land}*w_{il-bai3}\;+V_{Grass\;land}*w_{il-bai4}+V_{Bare\;land}*w_{il-bai5}\right)}_{time}$$

(15) $$I_{max-VCI}=\underset{time}{\max}{\left(V_{Cultivated\;land}*w_{il-vci1}+V_{Grass\;land}*w_{il-vci2}+V_{Bare\;land}*w_{il-vci3}\right)}_{time}$$

(16) $$I_{max-NGF}=\left[\underset{time}{\max}{\left(V_{Precipitation}\right)}_{time},\underset{time}{\max}{\left(V_{DEM}\right)}_{time},\underset{time}{\max}{\left(V_{Vegetation\;coverage}\right)}_{time}\right]$$

(17) $$I_{max-WDI}=\underset{time}{\max}{\left(V_{Surface\;runoff}\right)}_{time}$$

where time represents the different period.

**Figure 4:**
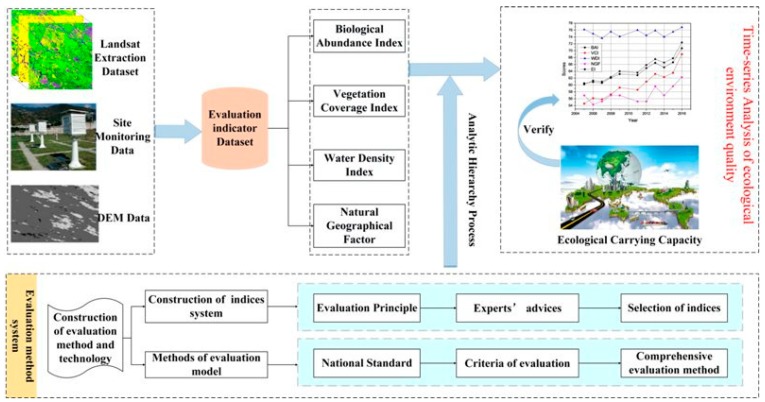
Flow chart of quantitative evaluation of the ecological environment.

Finally, the ecological carrying capacity based on the ecological footprint is applied in order to verify the effectiveness of the evaluation model. The flow chart of quantitative evaluation of the ecological environment is shown in Figure 4.

## 4. Results and Discussion

### 4.1. Index Analysis

The eco-environment evaluation model for the mining area is based on HJ/T192-2006. The weights of criterion layer and index layer are calculated to make the evaluation model achieve the most suitable trade-off using Equations (1)–(6) and the results are shown in Table 7.

**Figure 5:**
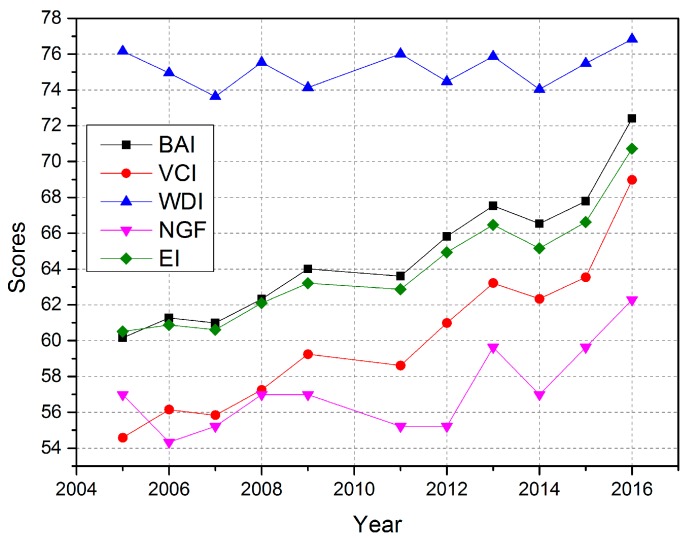
Scores of the EI and the sub-indices from 2005 to 2016.

Combined with the BAI, VCI, WDI, and NGF, the EI is calculated based on AHP. The scores of the different indices are shown in Figure 5 and Table 8.

Since the integration of the state’s coal mining enterprises in Yuxian County in 2008, small-scale coal enterprises have been closed down or merged with large state-owned coal enterprises. The BAI and the VCI of the mining area showed slow growth on the whole (Figure 5). Vegetation growth was restrained in the year of 2014 in the mining area. Meanwhile, the WDI was also relatively low. The WDI was mainly used to characterize the abundance of water resources in study area, and was thus affected by precipitation and groundwater. The WDI fluctuated between 73–77, and the variation range was relatively small.

The NGF was influenced by vegetation coverage, DEM, precipitation, etc. Overall, the NGF was mainly influenced by the slope of the study area, because of the mining causing ground collapse and landslides. An undulating terrain formed as a result of these events. The NGF was mainly influenced by DEM and precipitation from 2008 to 2012. In 2014, the NGF was affected by precipitation and vegetation coverage. The BAI and the VCI show similar trends to the EI in Figure 5.

**Figure 6:**
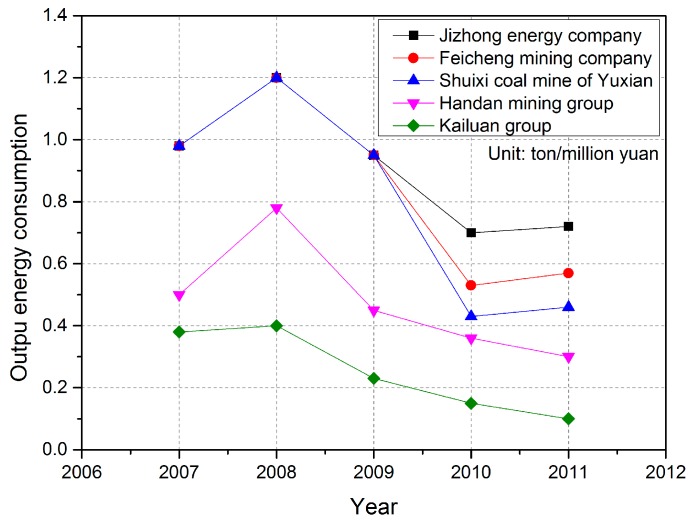
Output energy consumption of the coal mining enterprises in Yuxian.

Since the “7.14” coal mine disaster that occurred in Yuxian in 2010, which claimed the lives of 35 coal mine workers, China has begun to integrate coal mining enterprises. Shutting down small-scale coal mining enterprises, restructuring, and technological innovation have been put into effect. Figure 6 shows the output energy consumption of Yuxian before rectification and after rectification. The output energy consumption of each mineral company, especially the Kailuan Group, decreased with time. Energy consumption index represents that how much energy would be consumed for unit production value. The bigger value the index represents, the more coal would be consumed, which bring on more environment pollution. Based on Figure 5 and Figure 6, it could be found that there is a negative correlation between EI and energy consumption index. When the energy consumption index becomes higher, the EI will present a downward trend in the next two years, vice versa. The Cuijiazhai mining area and the Shanhou mining area are the part of the Kailuan Mining Bureau. Technological innovation and improvement not only promote economic development, but also reduce energy consumption and has important significance for the restoration and management of the eco-environment.

**Figure 7:**
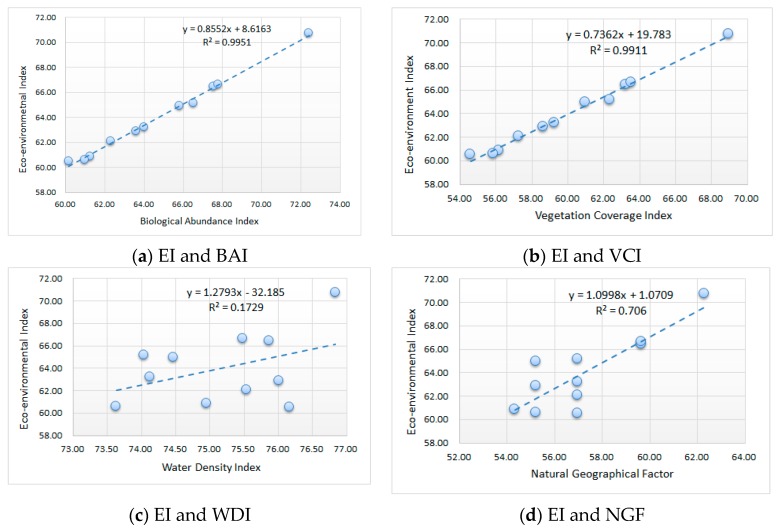
Correlation coefficients between the EI and the sub-indices. (**a**) the correlation coefficients between the EI and BAI; (**b**) the correlation coefficients between the EI and VCI; (**c**) the correlation coefficients between the EI and WDI; (**d**) the correlation coefficients between the EI and NGF.

The correlation coefficients were calculated between the EI and the sub-indices in order to find the driving factors of the EI. The correlation coefficients are shown in Figure 7.

The R^2^ values between the EI and BAI/VCI are 0.9951 and 0.9911, respectively, which indicates that the BAI and the VCI are both driving factors of the EI. However, the BAI and the VCI were calculated by land cover in the study area, and there is sufficient evidence to say that environmental quality is mainly determined by land-cover types in the Yuxian coalfield. The next decisive factor is NGF, with R2 equal to 0.7060. The correlation between the EI and the WDI is not significant, with R^2^ equal 0.1729, and the reason for this is the precipitation. The ground runoff fluctuates with the change of precipitation. The precipitation does not have a fixed regular pattern in the warm temperate continental monsoon climate, so the ground runoff is also irregular.

### 4.2. Environmental Quality Analysis and Validation

The EI has shown an increasing trend, as a whole, and this phenomenon has occurred since 2008. However, there was an exception, the EI declined in 2014. The reason for this may be the growth of vegetation was blocked in 2014. Wang et al. [52] analyzed the temporal and spatial characteristics of the NPP of the Yuxian coalfield between 2013 and 2015, based on high-resolution remote sensing data, and their results showed that the NPP value was the lowest in 2014.

The final results are divided into different levels to evaluate the quality of the eco-environment. The five levels of eco-environmental quality and four levels of change degree are divided based on HJ/T192-2006. The details are shown in Table 9 and Table 10. According to the rank of the eco-environmental quality, the environmental condition of the study area is ‘good’.

On the other hand, the ecological carrying capacity calculated based on the ecological footprint was used to verify the result of the evaluation model. Based on the land utilization in the mining area, we obtained data such as the area of cultivated land, area of grassland, area of building land, area of productive land, area of water, and area of bare land, from 2005 to 2016, which is shown in Table 11.

These data were divided by the total population of the study area and then multiplied by the corresponding balance factors and yield factors. The computational results were accumulated to obtain the per capita ecological carrying capacity of the coalfield in the study area. Based on the suggestions for computation of the ecological carrying capacity made by the World Commission on Environment and Development [54], 12% should be deducted from the ecological carrying capacity as the productive area for biological protection.

**Figure 8:**
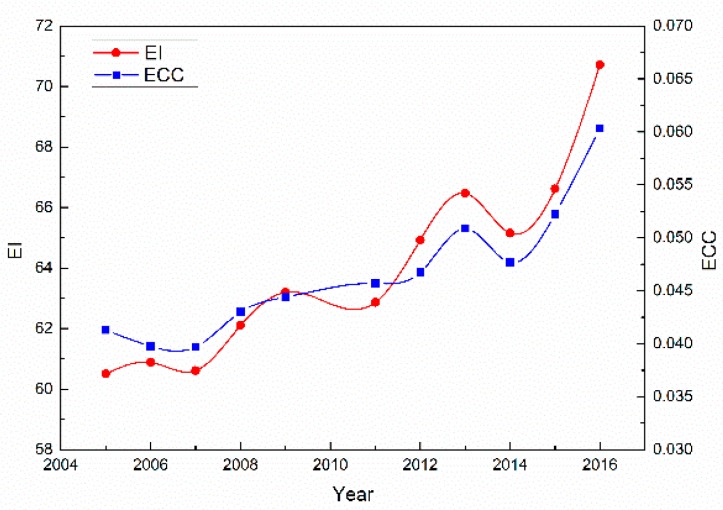
The trend of the EI and ecological carrying capacity over 2005 to 2016.

**Figure 9:**
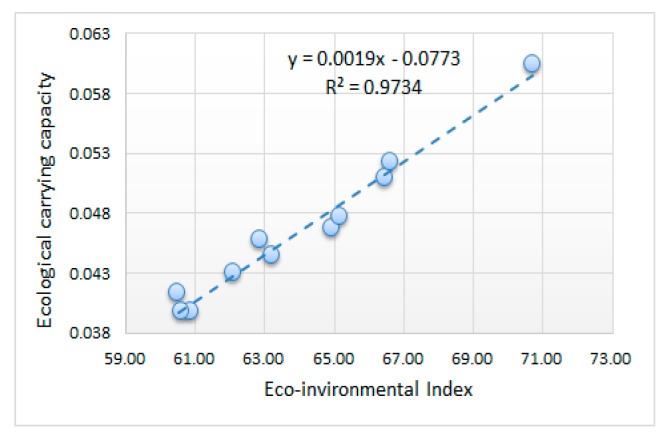
The correlation coefficients of the EI and ecological carrying capacity.

The ecological carrying capacity, which is shown in Table 12, Figure 8 and Figure 9, shows a similar trend to the EI. Because of the ground collapse caused by the mining, a large amount of land was fully utilized. The reason for the low ecological carrying capacity is the large population and the mining activities in the area. The per capita ecological carrying capacity increased from 0.0413 (hm^2^/cap) to 0.0604 (hm^2^/cap) between 2005 and 2016, after deducting 12% of the productive area for biological protection. This demonstrates that the ecological carrying capacity is low. The EI increased from 66.62 to 70.71 between 2015 and 2016, which was relatively large. It is shown in Table 12 that the eco-environment quality was slightly improved between 2015 and 2016. According to the reports of government work in Yuxian County, the total grain output of Yuxian County was 55,624 ton in 2010 and it almost tripled in 2016, reaching 145,800 ton. Besides, since Zhangjiakou City, which is the superior administrative agency of Yuxian County, was elected as the host city of XXIV Olympic Winter Games in July 2015, a series of greening actions began to be implemented and seventy-five square kilometers of fine greening project was completed in 2016. This can also serve as evidence that the improvement of EI in 2016 was relatively large.

The correlation coefficients between ecological carrying capacity and the EI were calculated in order to verify the effectiveness of the evaluation model. The fitting curve of the EI and ECC, with high R^2^ values, indicates that the EI and ECC are closely correlated. This phenomenon also illustrates that the results of the novel evaluation model based on multi-temporal remote sensing images and auxiliary data are acceptable.

Although environmental quality has shown an improving trend from 2005 to 2016, the protection and restoration of the eco-environment in the Yuxian coalfield is still an urgent problem that needs to be solved. The eco-environment of the coalfield had been seriously affected by the large-scale and heavy exploitation of the coal resources during the past decades. This damage has a long-term influence and is difficult to recover. The EI has become better in recently years because of the introduction of artificial greening project and the adequate precipitation. Tiny fractures in rock that can store water were collapsed during periods of coal mining. And then the surface vegetation growth would rely on the external water sources and human disturbance. When WDI dropped in 2014, EI also declined. The aquifer restoration requires long-term efforts. In recent years, the government and relevant departments have become aware of the importance of ecological restoration of coal mining areas, and some measures have been taken to control and improve the situation. However, if these problems are not paid enough attention, environmental pressure will accumulate because of the exploitation of the coal resources. Moreover, species diversity will decrease and the human living environment may be threatened in the mining area. Ultimately, the eco-environment will not be able to adapt to the development of the economy and society.

## 5. Conclusions

This paper provides a quantitative evaluation of the eco-environmental quality of the Yuxian coalfield, based on land-cover and statistical data from 2005 to 2016, using remote sensing and a multi-criteria decision-making technique. The land cover was utilized as the basic indicator for eco-environmental analysis of mining area with a small number of auxiliary indicators. What’s more, the weights are calculated to make the evaluation model achieve the most suitable trade-off by AHP. The mine-areas-oriented model is more suitable than the recommended indicators and weights given by HJ/T 192-2006. To verify the validity of our model, the ecological carrying capacity based on the ecological footprint is introduced. The eco-environmental quality of the Yuxian coalfield is not in an optimal state of harmonious development, based on the computed results. Although the EI has shown a slowly increasing trend, the value of the EI is low, as a whole, and the level of eco-environmental quality is only ‘good’. The BAI and the VCI are driving factors which have a good correlation with the EI. It can be seen from Table 9 that the per capita ecological carrying capacity increased to 0.0604 (hm^2^/cap) in 2016 from 0.0413 (hm^2^/cap) in 2005, which is consistent with the EI, and the R^2^ is 0.9734. By combining features extracted from remoting sensing and auxiliary data, the comprehensive evaluation model of the mining area in Yuxian County was established, achieving a quantitative evaluation of the eco-environment of the mining area of Yuxian County.

In conclusion, the research results could provide a basis for the management of the eco-environment of the Yuxian mining area, and a reference and scientific basis for achieving sustainable development of the eco-environment in the coalfield. The evaluation method is a supplement to the time-series quantitative evaluation of the eco-environment, and also helps us to explore the eco-environment in the mining area.

## Figures and Tables

**Table 1:** The remote sensing data used in this study.

Sensor	Date	Sensor	Date	Sensor	Date
ETM+	2005/04/03	ETM+	2008/11/21	OLI	2013/09/15
ETM+	2005/06/22	ETM+	2009/02/09	OLI	2013/11/18
ETM+	2005/09/17	ETM+	2009/06/24	OLI	2014/03/10
ETM+	2005/11/19	ETM+	2009/09/21	OLI	2014/07/25
ETM+	2006/03/05	ETM+	2009/10/23	OLI	2014/09/27
ETM+	2006/06/16	ETM+	2011/05/22	OLI	2014/11/21
ETM+	2006/09/29	ETM+	2011/07/06	OLI	2015/03/13
ETM+	2006/10/31	ETM+	2011/09/11	OLI	2015/08/13
ETM+	2007/02/20	ETM+	2011/11/14	OLI	2015/09/14
ETM+	2007/08/15	ETM+	2012/02/18	OLI	2015/11/01
ETM+	2007/09/23	ETM+	2012/07/02	OLI	2016/03/24
ETM+	2007/11/03	ETM+	2012/08/28	OLI	2016/08/06
ETM+	2008/03/10	ETM+	2012/10/06	OLI	2016/08/31
ETM+	2008/07/23	ETM+	2013/03/15	OLI	2016/11/19
ETM+	2008/09/02	OLI	2013/07/06	_____	_____

**Table 2:** The accuracy assessment for the image on 2016.

Scene	Category	Building Land	Water Body	Cultivated Land	Grassland	Bare Land
2016/03/24	Building land	108	0	0	0	0
	Water body	0	65	0	0	0
	Cultivated land	0	0	57	0	0
	Grassland	1	1	0	322	13
	Bare land	0	0	0	4	112
	User’s accuracies	1	1	1	0.9555	0.9655
	Producer’s accuracies	0.9908	0.9848	1	0.9877	0.896
	Commission	0	0	0	0.0445	0.0345
	Omission	0.0092	0.0152	0	0.0123	0.104
2016/08/06	Building land	108	0	0	0	0
	Water body	0	65	0	0	0
	Cultivated land	0	0	57	0	0
	Grassland	1	1	0	322	13
	Bare land	0	0	0	4	112
	User’s accuracies	1	1	1	0.9555	0.9655
	Producer’s accuracies	0.9908	0.9848	1	0.9877	0.896
	Commission	0	0	0	0.0445	0.0345
	Omission	0.0092	0.0152	0	0.0123	0.104
2016/08/31	Building land	108	0	0	0	0
	Water body	0	65	0	0	0
	Cultivated land	0	0	57	0	0
	Grassland	1	1	0	322	13
	Bare land	0	0	0	4	112
	User’s accuracies	1	1	1	0.9555	0.9655
	Producer’s accuracies	0.9908	0.9848	1	0.9877	0.896
	Commission	0	0	0	0.0445	0.0345
	Omission	0.0092	0.0152	0	0.0123	0.104
2016/11/19	Building land	108	0	0	0	0
	Water body	0	65	0	0	0
	Cultivated land	0	0	57	0	0
	Grassland	1	1	0	322	13
	Bare land	0	0	0	4	112
	User’s accuracies	1	1	1	0.9555	0.9655
	Producer’s accuracies	0.9908	0.9848	1	0.9877	0.896
	Commission	0	0	0	0.0445	0.0345
	Omission	0.0092	0.0152	0	0.0123	0.104

**Table 3:** Computing methods of all indicators in index layer.

Index Layer	Computing Method
V_Building land_	Area of building land/Total area
V_Water body_	Area of water body/Total area
V_Cultivated land_	Area of cultivated land/Total area
V_Bare land_	Area of bare land/Total area
V_Grass land_	Area of grass land/Total area
V_Vegetation coverage_	(Area of grass land + Area of cultivated land)/Total area
V_Precipitation_	The value of precipitation
V_DEM_	The value of DEM
V_Surgace runoff_	The value of water body

**Table 4:** The importance of the pair-wise comparison judgment [45].

Numerical Rating	Verbal Judgment of Preference
1	Equally preferred
3	Moderately preferred
5	Strongly preferred
7	Very strongly preferred
9	Extremely strongly preferred
2, 4, 6, 8	The adjacent middle value judgment
inversion	Comparison of factor i to j is b, while the factor j to i comparison scale is 1/b

**Table 5:** The standard values of the ratio index [30].

Number of Indices	2	3	4	5	6	7	8	9	10
RI	0	0.58	0.90	1.12	1.24	1.32	1.41	1.45	1.49

**Table 6:** Description of the land-cover types and parameters.

Land Type	Main Purpose	Balance Factor	Yield Factor
Cultivated land	Crop cultivation	1.10	0.91
Forest land	Providing forest products and wood	0.67	0.69
Grassland	Providing animal by-products	0.48	1.55
Building land	Land for human settlement	1.10	1.55
Productive water	Providing aquatic products	0.38	0.91
Energy land	Absorbing CO_2_ released by humans	0.67	0.00

**Table 7:** Evaluation index system.

Target Layer	Criterion Layer	Criterion Weight	Index Layer	Index Weight
Eco-environmental Index	Biological abundance index	0.46	Water body	0.42
			Building land	0.10
			Cultivated land	0.16
			Grassland	0.26
			Bare land	0.06
	Vegetation coverage index	0.26	Cultivated land	0.32
			Grassland	0.56
			Bare land	0.12
	Natural geographical factors	0.14	Precipitation	0.19
			DEM	0.28
			Vegetation coverage	0.58
	Water density index	0.14	Surface runoff	1.00

**Table 8:** The statistical results of the EI and the sub-indices.

Year	BAI	VCI	WDI	NGF	EI
2005	60.16	54.58	76.16	56.98	60.50
2006	61.27	56.15	74.95	54.33	60.88
2007	60.99	55.83	73.63	55.21	60.61
2008	62.32	57.25	75.53	56.98	62.10
2009	64.00	59.25	74.13	56.98	63.20
2011	63.60	58.61	76.02	55.21	62.87
2012	65.82	60.98	74.47	55.21	64.93
2013	67.53	63.21	75.87	59.63	66.47
2014	66.53	62.33	74.03	56.98	65.15
2015	67.78	63.54	75.48	59.63	66.62
2016	72.40	68.97	76.84	62.28	70.71

**Table 9:** Grade of eco-environmental quality [53].

Level	Excellent	Good	General	Poor	Bad
Value range	EI≥ 75	55≤ EI ≤ 75	35≤ EI ≤ 55	20≤ EI ≤ 35	EI ≤ 20
State	Vegetation coverage and greenness is excellent; the ecosystem is suitable for human survival	Vegetation coverage and greenness is good; the ecosystem is suitable for human survival	Vegetation coverage and greenness is average; the ecosystem is suitable for human survival but there are factors that are not suitable for human survival	Vegetation coverage is poor; severe drought; fewer species; there are obvious factors that are not suitable for human survival	The situation is bad; desert, saline or alpine region all around; environmental degradation

**Table 10:** Grade of variation of eco-environmental quality (HJ/T192-2006).

Level	No clear Change	Slight Change	Clear Change	Significant Change
Change value	|ΔEI|≤2	2<|ΔEI|≤5	5<|ΔEI|≤10	|ΔEI|>10
State	There is no clear change in the ecological environment	If 2<ΔEI≤5, the eco-environment is slightly better, if −2>ΔEI≥−5, the eco-environment is slightly worse.	If 5<ΔEI≤10, the eco-environment is clearly better, if −5>ΔEI≥−10, the eco-environment is clearly worse.	If ΔEI>10, the eco-environment is significantly better, if −ΔEI<−10, the eco-environment is clearly worse.

**Table 11:** Computational results of the per capita area of the study area.

Land Type		Cultivated Land	Grassland	Water Body	Building Land	Total
Per capita area (hm^2^/cap)	2005	0.0158	0.0210	0.0064	0.0116	0.0549
	2006	0.0097	0.0263	0.0063	0.0122	0.0545
	2007	0.0098	0.0259	0.0062	0.0124	0.0542
	2008	0.0115	0.0271	0.0063	0.0134	0.0584
	2009	0.0118	0.0305	0.0062	0.0123	0.0608
	2011	0.0153	0.0277	0.0064	0.0123	0.0616
	2012	0.0123	0.0331	0.0066	0.0122	0.0643
	2013	0.0156	0.0353	0.0064	0.0122	0.0695
	2014	0.0103	0.0361	0.0062	0.0133	0.0659
	2015	0.0147	0.0358	0.0063	0.0142	0.0711
	2016	0.0158	0.0434	0.0064	0.0167	0.0823

**Table 12:** Computational results of the per capita ecological carrying capacity of the study area.

Land Type		Cultivated Land	Grassland	Water Body	Building Land	Total
Per capita ecological carrying capacity (hm^2^/cap)	2005	0.0139	0.0138	0.0033	0.0103	0.0413
	2006	0.0085	0.0172	0.0033	0.0107	0.0398
	2007	0.0086	0.0169	0.0032	0.0109	0.0397
	2008	0.0102	0.0178	0.0033	0.0118	0.0430
	2009	0.0104	0.0199	0.0032	0.0108	0.0444
	2011	0.0134	0.0181	0.0033	0.0108	0.0457
	2012	0.0108	0.0217	0.0034	0.0108	0.0467
	2013	0.0137	0.0231	0.0033	0.0107	0.0509
	2014	0.0091	0.0236	0.0032	0.0118	0.0477
	2015	0.0146	0.0263	0.0037	0.0141	0.0522
	2016	0.0139	0.0284	0.0033	0.0147	0.0604
